# Encapsulation of Polyphenols from *Lycium barbarum* Leaves into Liposomes as a Strategy to Improve Their Delivery

**DOI:** 10.3390/nano11081938

**Published:** 2021-07-28

**Authors:** Ramona-Daniela Păvăloiu, Fawzia Sha’at, Georgeta Neagu, Mihaela Deaconu, Corina Bubueanu, Adrian Albulescu, Mousa Sha’at, Cristina Hlevca

**Affiliations:** 1 National Institute for Chemical-Pharmaceutical Research & Development—ICCF Bucharest, Vitan Av. nr. 112, 3rd District, 031299 Bucharest, Romania; fawzya.shaat@gmail.com (F.S.); georgetaneagu2008@gmail.com (G.N.); cbubueanu@gmail.com (C.B.); rockady2@gmail.com (A.A.); crhlevca@gmail.com (C.H.); 2 Department of Inorganic Chemistry, Physical-Chemistry & Electrochemistry, Faculty of Applied Chemistry and Materials Science, University “Politehnica” of Bucharest, Gheorghe Polizu Street nr. 1–7, 1st District, 011061 Bucharest, Romania; mihaelladeaconu@gmail.com; 3 Department of Molecular Virology, Stefan S. Nicolau Institute of Virology, Mihai Bravu Av. nr. 285, 3rd District, 030304 Bucharest, Romania; 4 Department of Pharmaceutical Technology, Faculty of Pharmacy, University of Medicine and Pharmacy Grigore T. Popa, Universitatii Street. nr. 16, 700115 Iasi, Romania; mousa.shaat1@gmail.com

**Keywords:** cytoprotective effect, liposomes, polyphenols release, *Lycium barbarum*

## Abstract

This study is focused on the encapsulation of polyphenols from *Lycium barbarum* leaves into liposomes as a strategy to improve their delivery. Liposomes loaded with *Lycium barbarum* leaves extract were obtained and characterized for particle size, polydispersity, entrapment efficiency, and stability. Liposomes presented entrapment efficiency higher than 75%, nanometric particle size, narrow polydispersity, and good stability over three months at 4 °C. The liposomes containing *Lycium barbarum* offered a slower release of polyphenols with attenuated burst effect compared with the dissolution of free *Lycium barbarum* extract in phosphate buffer solution at pH 7.4. Moreover, an in vitro pretreatment of 24 h with loaded liposomes showed a cytoprotective effect against H_2_O_2_-induced cytotoxicity on L-929 mouse fibroblasts cells. These preliminary findings imply that liposomes could be successfully employed as carriers for polyphenols in pharmaceutical applications.

## 1. Introduction

*Lycium barbarum* (fam. Solanaceae), known as goji or wolfberry, is widely grown in the subtropical areas of the world, in Japan and Korea, in countries from south-east of Asia, as well as in Europe. In the last years, Goji berries have been used in Chinese herbal medicine and have drawn considerable attention thanks to their diverse biological activities, such as being a liver and kidney protector and eyesight enhancer [1]. A large part of the studies on *L. barbarum* have mainly focused on plant fruit (Goji berries), used as a food product [2]. Moreover, medicinal research has shown that *L. barbarum* extract (leaves and fruit) has immune-modulator, antioxidant, anti-aging, anticancer, hypotensive, and hypoglycemic properties. All these beneficial effects are dependent on the content of polyphenols, polysaccharides, flavonoids, carotenoids, nitrogen-containing compounds, and other constituents, which are used to promote good health [3,4]. However, the compounds from *L. barbarum* extract have certain limitations such as poor solubility, toxicity, and degradation in a gastrointestinal medium, which can lead to a low level of plasma concentrations of compounds and an inefficient therapeutic effect.

A way to reduce these limitations is the encapsulation of *L. barbarum* extract into innovative drug delivery systems, such as liposomes, inorganic nanoparticles, polymeric nanoparticles, micro or nanoemulsions, and so on [5,6,7]. Liposomes have received much attention as one of the most promising vehicles for the incorporation of bioactive substances thanks to their remarkable characteristics: biocompatibility, biodegradability, non-immunogenicity, non-toxicity, and the ability to incorporate hydrophilic and hydrophobic compounds. Therefore, these unique features make liposomes capable of enhancing the therapeutic activity of bioactive compounds by increasing solubility, and thus improving bioavailability, tailoring pharmacokinetics, increasing intracellular uptake, and providing better stability [8,9]. Several papers have confirmed the role of liposomes as delivery systems for compounds from natural sources, such as quercetin [10], silymarin [11], curcumin [12], resveratrol [13], rutin [14], colchicine [15], catechins [16], breviscapin [17], and essential oils (*Artemisia arborescens* L. [18], chamomile [19]), as well as plant extracts, such as *Polygonum aviculare* L. [20], *Glycyrrhiza glabra* L. [21], *Platycladus orientalis* L. [22], and *Hibiscus sabdariffa* L. [23].

A small number of papers have reported the encapsulation of bioactive compounds from *L. barbarum* into nanoemulsions, liposomes, and nanoparticles. For instance, Bo et al. [24] designed liposomes loaded with polysaccharides from *L. barbarum* berries with good efficiency and nanosize that effectively enhanced the process of phagocytosis of peritoneal macrophages by inducing the production of nitric oxide in mouse macrophage cells. Zu et al. [25] obtained monodisperse and nano-size *L. barbarum* lipid-derived nanoparticles with great cellular uptake by macrophages, as well as a significant anti-inflammatory effect. Oral administration of *L. barbarum* lipid-derived nanoparticles provided desirable therapeutic efficacy against ulcerative colitis. Zhang et al. [26] reported the incorporation of a compound extracted from *L. barbarum* berries, zeaxanthin, into nanocapsules with high homogeneity and enhanced thermal stability by complex coacervation with gelatin and sodium carboximetilcelulose. Luo et al. [27] produced nanoemulsions containing carotenoid esters from *L. barbarum* berries that demonstrated higher liberation and bioaccessibility than free carotenoid esters. All these studies addressed *L. barbarum* berries, as vegetal material, and from our knowledge, no previous papers related to the encapsulation of leaves extracts into liposomes were reported.

Our work was focused on developing a delivery system based on liposomes for *L. barbarum* leaves extract to improve efficiency. To fulfill this objective, liposomes loaded with *L. barbarum* extract were obtained by the film hydration method combined with sonication and extrusion. It was investigated whether the pretreatment of 1 h or 24 h with loaded liposomes affected the L-929 mouse fibroblasts cells cytotoxicity induced by H_2_O_2_. Moreover, the features of liposomes loaded with *L. barbarum* as particle size, polydispersity index (PDI), entrapment efficiency (EE), stability, and polyphenols’ release from liposomes were analyzed, and all the data are reported herein.

## 2. Materials and Methods

### 2.1. Materials

Phosphatidylcholine (egg yolk), 2,2-diphenyl-1-picrylhydrazyl (DPPH), Folin–Ciocalteu reagent, sodium cholate, sodium carbonate, Triton X-100, a mixture of penicillin, neomycin, streptomycin dissolved in 0.9% NaCl (PSN), L-ascorbic acid, hydrogen peroxide 30% (*w*/*w*) (H_2_O_2_), PBS, fetal equine serum (FES), Eagle’s minimum essential medium (EMEM), and 0.25% trypsin-ethylene-diamine-tetraacetic-acid (EDTA) solution were acquired from Sigma-Aldrich (Darmstadt, Germany). High-performance liquid chromatography (HPLC) standards, catechin hydrate, (-)epicatechin, rutin hydrate, kaempferol, myricetin, quercetin, and trans-resveratrol, as well as caffeic, chlorogenic, *p*-coumaric, gallic, rosmarinic, protocatechuic, caftaric, vanillic, syringic, trans-ferulic, ellagic dihydrate, and chicoric acids, were acquired from Sigma-Aldrich (Darmstadt, Germany). Rhodamine B from Sigma-Aldrich (Darmstadt, Germany), WGA-AlexaFluor 488 conjugate from Thermo Fischer (Waltham, Massachusetts State or Province, US), and Hoechst 33342 from Molecular Probes (Eugene, Oregon, US) were used as fluorophores. The murine fibroblast cell line (L-929) was obtained from ATCC^®^CRL-6364™ (Manassas, Virginia, US) and the CellTiter96^®^aqueous non-radioactive cell proliferation assay was purchased from Promega (Madison, WI, USA).

### 2.2. Plant Material and L. barbarum Extract Preparation

Leaves of spontaneously growing *L. barbarum* were harvested from Dambovita, Romania, Europe (44°53′27.4′′ N 25°28′28.4′′ E). The taxonomic identification was made by the botanical specialists of INCDCF-ICCF Bucharest, Romania. A voucher specimen was stored in INCDCF-ICCF Plant Material Room, Romania.

The *L. barbarum* leaves were dried at 25 °C and milled into a fine powder. Twenty-four grams (24 g) of vegetal powder were extracted with a hydro-alcoholic solution (ethanol 50% *v/v*), 1/10 plant/solvent ratio (*m/v*) at reflux temperature for 1 h under continuous stirring. The suspension was filtered to remove plant waste and evaporated at a temperature of 60 °C and reduced pressure (72–75 mm Hg), and then re-dissolved in hydro-alcoholic solution. The extraction yield was 10.83%. The *L. barbarum* extract obtained with a concentration of 21.67 mg/mL was kept at 5 °C until analysis.

### 2.3. Characterization of L. barbarum Extract

*L. barbarum* leaves extract was characterized concerning its total phenolic content and chemical composition. The total phenolic content of *L. barbarum* extract was assessed by Folin–Ciocalteu assay [28]. According to this method, an aliquot (1 mL *L. barbarum* extract), 10 mL of water, and 1 mL of Folin–Ciocalteu reagent (1:10 dilution) were mixed. After homogenization, a 5% sodium carbonate aqueous solution (*w/v*) was added to complete the 25 mL final volume. Samples were maintained at 25 °C for 30 min. The samples were measured at 760 nm absorbance, using a UV/VIS spectrophotometer (Jasco V-630, Portland, OR, USA). Total phenolic content was calculated from the extrapolation of the gallic acid calibration curve (concentration range: 0.01–1 mg/mL; y = 0.01322x + 0.0272; correlation coefficient R^2^ = 0.99574) and expressed as milligram gallic acid equivalents (mg GAE)/g plant material. Quantitative and qualitative analysis of *L. barbarum* leaves extract was performed using HPLC as described by Pavaloiu et al. [29]. Phenolic compounds of *L. barbarum* extract were identified by comparison with standards (Table S1, Supplementary Information).

### 2.4. Preparation of Liposomes Loaded with L. barbarum Extract

Liposomes loaded with *L. barbarum* (coded LB_L1 and LB_L2) were obtained by the film hydration method combined with sonication and extrusion. There were two lipid solutions prepared: (i) phosphatidylcholine and *L. barbarum* extract (8:2.5 *w/w*); and (ii) phosphatidylcholine, *L. barbarum* extract, and sodium cholate (8:2.5:2 *w/w*) dissolved in 5 mL of methanol. Subsequently, the lipid samples were maintained at 25 °C for one night to ease the swelling of the phosphatidylcholine. The lipid samples were evaporated (Laboranta 4000 Rotary evaporator, Heidolph Instruments GmbH & Co. KG, Kelheim, Germany) at vacuum for 2 h at 37 °C. After the complete removal of the solvent, the resulting thin lipid films were hydrated with distilled water at 37 °C. The obtained suspensions were maintained for 2 h at 25 °C to produce stabilized liposomes. The liposome size was decreased through sonication and extrusion. The sonication process was performed with an ultrasonic bath (Sonorex-Digital-10P, Bandelin-Electronic, Berlin, Germany) for 25 min and power delivery controlled as percentage amplitude was set at 20%. The extrusion was carried out stepwise: first using a filter with a pore size of 0.4 μm and then 0.2 μm; for each pore size, five extrusion cycles were carried out. The lipid samples were centrifuged at 10,000 rpm and 5 °C for 25 min to separate the loaded liposomes from the free extract; the supernatant was removed in a controlled manner and the sediment containing liposomes loaded with *L. barbarum* was redispersed in water. Empty liposomes (coded L1 and L2) were used as controls. All lipid samples were prepared in triplicate and kept at a temperature of 5 °C.

### 2.5. Characterization of Liposomes

The liposomes loaded with *L. barbarum* were characterized in terms of the EE, PDI, and particle size. The EE was calculated as the ratio between the quantity of polyphenols that were entrapped in the liposomes and the total quantity of polyhenols in the *L. barbarum* leaves extract. The quantity of total polyphenols entrapped in liposomes was assessed by the Folin–Ciocalteu method described in several papers [30,31]. To further identify and quantify the polyphenols entrapped in the liposomes, the supernatants were analyzed through HPLC, as described in Section 2.3. The quantification of polyphenols loaded in liposomes was assessed indirectly considering that the supernatants contain the extract non-entrapped in liposomes.

The PDI and mean diameter of the liposomes were measured using a particle size analyzer (Beckman-Coulter-N4-PCS-Submicron, Paris, France) by the dynamic light scattering (DLS) technique. To avoid multiple scattering effects, an amount of distilled water was added to the liposomal dispersions; the dilution ratio was 1:10. All the measurements were carried out at a temperature of 25 °C using triplicates and ten runs for each measurement.

### 2.6. In Vitro Polyphenols Release Study from Liposome Formulations

The liposome formulations were subjected to a polyphenols release study using the dialysis membrane method with maintained sink conditions. The liposomal suspensions (1.0 mL) were transferred in dialysis bags with a 14,000 molecular weight cut-off. The bags with lipid samples were immersed in 50 mL PBS with a molar concentration of 0.1 M at pH 7.4. The experiments were performed at a temperature of 37 °C and a stirring rate of 100 rpm/min. Samples were taken (1 mL) at 0.25, 0.5, 0.75, 1, 2, 3, 4, 5, 6, 10, and 24 h with medium replenishment. The phenol content was determined by spectrophotometry and, for comparison, the free extract was also tested. The release studies were done in triplicate.

### 2.7. Free Radical Scavenging Activity

The antioxidant activity of the *L. barbarum* extract was determined using the Sanchez-Moreno et al. method [32]. Thus, DPPH methanolic solution of 0.025 g/L (2.950 mL) was added to 50 μL of each sample at different concentrations. The solutions were maintained for 30 min in the darkness at 25 °C. The absorbance sample was recorded at 517 nm using a UV/VIS spectrophotometer (Jasco V-630, Portland, Oregon, USA). Quercetin and caffeic acid were used as positive controls. Experiments for the antioxidant activity were done in five replicates.

The DPPH inhibition percentage was determined using the following Equation (1):

(1) $$\%inhibition=\frac{A_{0}-A_{sample}}{A_{0}}\times 100$$

where *A*_0_ represents the absorbance without sample (control) and *A_sample_* represents the sample absorbance.

### 2.8. In Vitro Cytotoxicity Assay

The cytotoxicity of *L. barbarum* was evaluated through the MTS method using CellTiter96^®^aqueous non-radioactive cell proliferation assay. The murine fibroblast cells were seeded on 96-well plates (7000 cells/well) (Manassas, VA, USA) for 24 h and then treated with 5, 10, 50, and 100 μg/mL of *L. barbarum* extract at a temperature of 37 °C, for one day. Cells incubated with medium, without plant extract, were used as a negative control. Then, the medium was removed, and a volume of 0.1 mL MTS/EMEM (1:10 *v*/*v*) was added and incubated for three more hours. The optical density was recorded at 490 nm using Microplate-Reader (Chameleon V Plate Reader, LKB Instruments) (Hidex, Corston, UK). The viability of cells is express as a percentage of negative control, set to 100% viability.

### 2.9. Oxidative Stress Induction Assay

The fibroblasts (L-929) were seeded on culture flasks for three days before in vitro studies to obtain an 80% confluence and placed on a 96-well microplate (1 × 10^5^ cells per well) (manufacturer, city, (State or Province), country). The oxidative stress was determined by exposure to 1, 10, 20, 50, and 100 mM of H_2_O_2_ solution in EMEM for 4 h.

To evaluate the effect of pretreatment with *L. barbarum*-loaded liposomes on the cytotoxicity induced by H_2_O_2_, L-929 cells were pretreated for 1 or 24 h with a nontoxic concentration of empty liposomes, free *L. barbarum* extract, and *L. barbarum*-loaded liposomes (5, 10, and 25 µg/mL, respectively). Afterward, the medium was replaced and 50 mM H_2_O_2_ was added to L-929 cells for another 24 h. As positive control, ascorbic acid was used. The viability of cells was determined by the MTS assay. The medium culture with and without H_2_O_2_ was considered as the control. The absorbance of each culture well was examined with Microplate-Reader (Chameleon V Plate Reader, LKB Instruments) (Hidex, Corston, UK) at 490 nm. All the samples used in these experiments were exposed for three hours to ultraviolet light for sterilization. Experiments for oxidative stress induction assay were performed in triplicate.

### 2.10. Uptake of Liposome by Fibroblasts

*L. barbarum*-loaded liposomes, L1_LB and L2_LB, labeled with Rhodamine (red fluorescence), were prepared to evaluate their internalization by fibroblast. L929 cells were cultivated for 24 h on cover slips and incubated for 24 h with liposomes, L1_LB and L2_LB, labeled with Rhodamine B (red fluorescence) in EMEM (100 µg mL^−1^). After washing with EMEM without phenol red, the cells were incubated for 30 min at 37 °C with WGA-AlexaFluor 488 conjugate (green fluorescence) (20 µg mL ^−1^, Thermo Fischer) for cell membrane staining. Washed two times with EMEM without phenol red, the cells were then incubated for 30 min at 37 °C with Hoechst 33342 (2 µg mL^−1^, Molecular Probes, Eugene, Oregon, US) for nucleic acid staining. WGA-AlexaFluor 488 conjugate and Hoechst solutions were freshly made in EMEM without phenol red. Every cover slip was washed two more times with EMEM without phenol red and mounted on microscopy slides. Images were acquired using a confocal microscope (Zeiss LSM710, Carl Zeiss, Jena, Germany) and Zeiss Zen v.10 software, version 2.3. (Carl Zeiss, Jena, Germany).

### 2.11. Statistical Analysis

All experimental studies were made at least in triplicate. Data were displayed as mean ± standard deviation (SD). Significant differences were considered at *p* < 0.05. The EC_50_ (the effective concentration that induces 50% DPPH inhibition) and the IC_50_ (the concentration that determines 50% reduction of the viable L-929 cells) were calculated using GraphPad Prism version 9.0.0.computer program (GraphPad Software, San Diego, CA, USA).

## 3. Results

### 3.1. Characterization of L. barbarum Extract

An ethanolic extract of leaves of *L. barbarum* was obtained and characterized for total phenolic content, quantitative chemical composition, and antioxidant and cytotoxic activity. The ethanolic extract from *L. barbarum* leaves showed a considerable value for polyphenols content of 18.30 ± 0.011 mg GAE/g plant material. Different values for spontaneously and cultivated growing *L. barbarum* were reported in various studies, e.g., 2.49 mg GAE/g dry material [33] and 61.59 mg GAE/g plant material [34]. All raw materials, those described in the above-mentioned studies [33,34], and plant material used in this paper were harvested from different counties of Romania, Europe. The differences regarding the polyphenols content between our results and the literature data are possibly explained by agro-climatic conditions like rainfall, altitude, or soil composition. Similar trends were described for other species in several papers [35,36].

**Figure 1:**
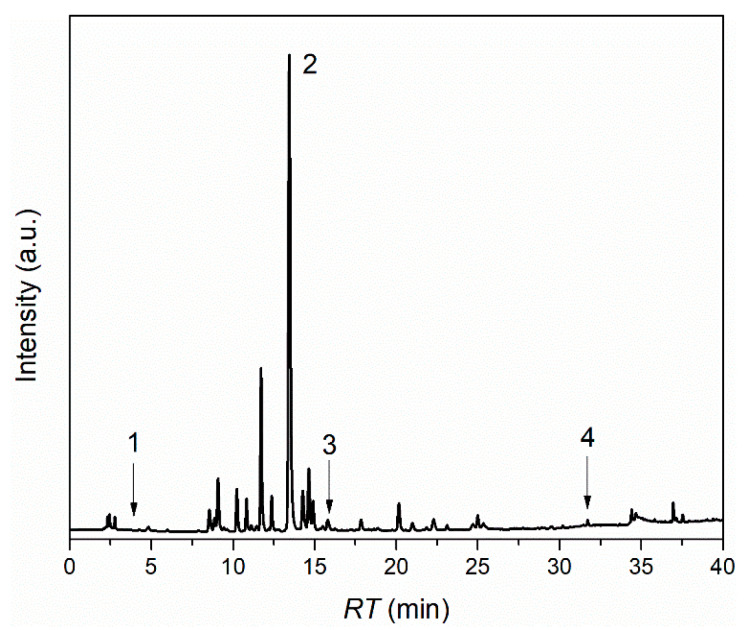
Chromatogram of *Lycium barbarum*; 1—gallic acid: RT = 3.62 min; c = 0.231 ± 0.002 mg/g extract; 2—chlorogenic acid: RT = 13.15 min; c = 18.740 ± 0.031 mg/g extract; 3—caffeic acid: RT = 14.48 min; c = 1.773 ± 0.002 mg/g extract; 4—rosmarinic acid: RT = 31.48 min; c = 0.108 ± 0.002 mg/g extract.

The composition of *L. barbarum* extract was analyzed with an HPLC system using a mixture of nineteen phenolic compounds. It can be observed in the chromatogram (Figure 1) that four compounds were detected and quantified. Chlorogenic acid (18.740 ± 0.031 mg/g extract) was presented in a significant amount in *L. barbarum* leaves extract. Caffeic acid was also detected at a concentration of 1.773 ± 0.002 mg/g. Furthermore, rosmarinic and gallic acids were also found, but in low concentrations: 0.108 ± 0.002 mg/g extract and 0.231 ± 0.002 mg/g extract, respectively. Our results are in accordance with the literature; for example, Mocan et al. showed that the chlorogenic and caffeic acids were components of *L. barbarum* leaves [33].

The cytotoxicity of *L. barbarum* on L-929 fibroblasts was assessed through the MTS assay. L-929 murine fibroblast cell is one of the recommended lines for the assessment of cytotoxicity by International Standard ISO-10993-5. *Biological evaluation of medical devices—Part 5. Tests for in vitro cytotoxicity,* and thus was used in this study. The cytotoxicity results are presented as the concentration that promotes a reduction of 50% in cell viability (IC_50_). The IC_50_ for the *L. barbarum* extract was 91.91 ± 0.292 µg/mL. In accordance with the U.S. National Cancer Institute Plant Screening Program, phytocompounds with IC_50_ measuring from 20 to 100 μg/mL are considered moderately cytotoxic [37,38]. Therefore, the IC_50_ value of *L. barbarum* extract represents a moderate cytotoxic activity of *L. barbarum* extract against the L-929 murine fibroblasts.

The capacity to induce DPPH inhibition was reported as the half-maximal effective concentration (EC_50_). *L. barbarum* extract had a significant antioxidant capacity (EC_50_ < 50 µg/mL). In comparison with controls, the EC_50_ of *L. barbarum* (EC_50_ = 11.33 ± 0.056 µg/mL) was around 2.6 and two times higher than EC_50_ of caffeic acid (4.370 ± 0.022 μg/mL) and quercitin (5.97 ± 0.026 μg/mL). Important antioxidant activity of *L. barbarum* was also reported in the literature (29.30 ± 4.34 μg quercitin equivalents/mg plant material [34]).

### 3.2. Characterization of Liposomes Loaded with L. barbarum

The liposomes loaded with *L. barbarum* extract were obtained by the film hydration method combined with sonication and extrusion. Afterward, these liposomes loaded with *L. barbarum* extract were characterized for EE, PDI, size, and stability over three months. Empty liposomes were used as controls. The characteristics of the liposomes are reported in Table 1.

The size of all samples was below 200 nm; empty lipid vesicles had smaller values (85.4 ± 0.341 nm for L1 and 105.2 ± 2.042 nm for L2), and the encapsulation of *L. barbarum* extract resulted in an enlargement in the size of particles (141.6 ± 2.360 nm for LB_L1 and 195.9 ± 1.220 nm for LB_L2). The *L. barbarum* extract has compounds with various polarities. Accordingly to Castangia et al. [21], their localization within liposomes is distinct: the lipid bilayer contains non-polar compounds, while the aqueous inner core contains polar compounds, with encapsulation of either one resulting in particle size enlargement. Moreover, the encapsulation of non-polar substances could induce a fluidization effect on the liposome membranes, resulting in a phospholipid bilayer disturbance through a profound insertion of phytocompounds. Our results are in agreement with several scientific articles. Gibis et al. [39] and Castangia et al. [21] stated the enlargement of particle size at the incorporation of grape-seed and liquorice extracts, respectively. The presence of sodium cholate (the surfactant) may cause a destabilization of phospholipids, thus leading to an increase in vesicle size thanks to its intercalation in the structure of phospholipid bilayer [40]. The formulation without sodium cholate (LB_L1) had a lower particle size than that with sodium cholate (LB_L2). All liposomal formulations showed small values of PDI (<0.2); these values confirm a lower tendency to form aggregates and good homogeneity of these colloidal systems.

The EE of *L. barbarum* extract in liposomes was 84.60 ± 2.23% for LB_L1 and 75.25 ± 1.761 for LB_L2 (reported in Table 1). These data showed that the preparation method was efficient, and that extract loss during preparation was minimal. Corresponding results are stated by Sinico et al. [18], Soon et al. [20], and Castangia et al. [21] for the encapsulation of *Artemisia arborescens* (EE = 74%), *Polygonum aviculare* (EE = 83%), and *Glycyrrhiza glabra* L. (EE = 84%), respectively. The formulation with sodium cholate (LB_L2) had smaller EE (%) than the one without sodium cholate (LB_L1); sodium cholate competed with plant extract for bilayer space. Thus, a smaller amount of plant extract can be incorporated into a phospholipid bilayer, leading to a reduction in EE, in agreement with Gupta et al. [40]. Moreover, in order to evaluate the composition of the polyphenols housed in the liposomes, an HPLC analysis was performed on supernatants obtained by centrifugation of lipidic dispersions after the extract was encapsulated in liposomes. The results of HPLC analysis performed on supernatants are presented in the Supplementary Information. Comparing the chromatogram of *L. barbarum* extract (Figure 1) with the chromatograms of LB_L1 and LB_L2 supernatants after encapsulation of *L. barbarum* extract (Figure S1; Supplementary Information), it was concluded that all four compounds that identified *L. barbarum* extract were encapsulated in liposomes. The quantity of an encapsulated polyphenol was calculated by subtracting the amount of that polyphenol used in the initial formulation from amount of that unbound polyphenol present in supernatant obtained after centrifugation of liposomes. Both nanoformulations contain large amounts of chlorogenic acid. Caffeic acid was completely encapsulated in liposomes without sodium cholate (LB_L1) and the formulation with cholate (LB_L1) present a large quantity of caffeic acid. The amount of gallic acid encapsulated in both types of liposomes was almost identical, as well as rosmarinic acid, which was completely encapsulated in both types of liposomes (Table S2, Supplementary Information).

A three-month stability study for liposomes loaded with *L. barbarum* was performed. The samples were stored at 4 °C in darkness to suppress the light-induced oxidation and hydrolysis of lipids. The stability of samples was evaluated by EE assessment at various storage times (up to three months). Liposomes loaded with *L. barbarum* were stable for the entire period of the study (three months), with almost the same quantity of plant compounds loaded after one month (phytocompound loss below 0.85%) and after three months (phytocompound loss below 5.00%). Moreover, the *L. barbarum*-loaded liposomes were visually stable, as no sedimentation was observed during storage. These data probably could be explained by higher Brownian motion and diffusion rates (resulting from liposomes) than the gravitational-induced sedimentation rate [41].

### 3.3. In Vitro Polyphenols Release Study

**Figure 2:**
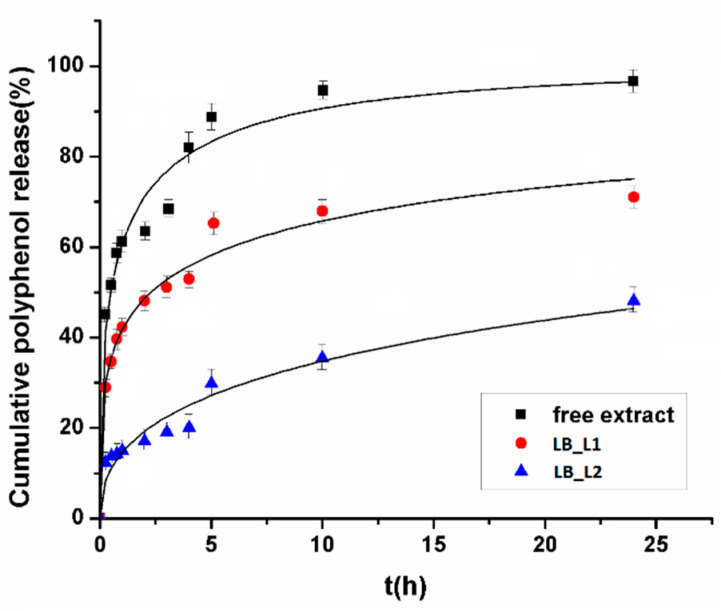
Polyphenols release from liposomes loaded with L. barbarum versus free L. barbarum fitted with Weibull function. LB_L1—liposomes loaded with L. barbarum; LB_L2—liposomes loaded with L. barbarum (formulation with sodium cholate).

The release of polyphenols from the liposomes loaded with *L. barbarum* extract and free extract were studied over a 24 h period in phosphate buffer solution at pH 7.4, a temperature of 37 °C, and a magnetic stirring of 150 rpm, and the polyphenols released were assessed by Folin–Ciocalteu assay. The results are presented in Figure 2. The dissolution of polyphenols profile from the free *L. barbarum* presented a “burst release” phenomenon (51.53 ± 0.28% was released in the initial 30 min); this phenomenon was attenuated by the encapsulation of the *L. barbarum* into liposomes (13.67 ± 0.18% were released in the initial half-hour from LB_L2 and 34.67 ± 0.15% from LB_L1). After ten hours, approximately all quantities of polyphenols from the free *L. barbarum* dissolute (96.62 ± 1.87%); meanwhile, liposomes produced a slower release, reaching 70.98 ± 3.12% for LB_L1 and 48.05 ± 3.50% for LB_L2 after 24 h.

The results were fitted by Weibull and Korsmeyer–Peppas models (Equations (2) and (3)), described by the following:

(2) $$\frac{M_{t}}{M_{\infty }}=1-\exp^{-at^{b}}$$

(3) $$\frac{M_{t}}{M_{\infty }}=k\cdot t^{n}$$

where *M_t_* is the cumulative quantity of polyphenols released at time *t; M∞* is the total polyphenols quantity; and *a, b, k*, and n are Weibull and Korsmeyer–Peppas parameters.

Table 2 presents the parameters of fitted release data. In the Weibull model, *a* indicates the rate of drug delivery and *b* shows the mechanism of release [42]. For all samples, values of *b* Weibull parameter are bellow 0.75, which indicates a Fickian diffusion. In the Korsmeyer–Peppas model, the *k* constant depends on the colloidal system features and the *n* parameter represents the diffusion exponent. The *n* parameter shows the nature of the release phenomenon. Thus, for *n* values of 0.5, polyphenols release shows a Fickian diffusion phenomenon; for *n* values of 1, the polyphenols release and time have a direct relationship; for *n* values between 0.5 and 1, polyphenols release indicates a non-Fickian diffusion (anomalous diffusion); and *n* values below 0.5 show a pseudo-Fickian diffusion. The diffusion exponent *n*, for all formulations, is below 0.5, indicating a pseudo-Fickian diffusion [42]. Other studies reported similar results, for example, the release profile of polyphenols of grape seed extract from lipid vesicles did not present any burst effect, and polyphenols transport followed a Fickian diffusion [23,43].

### 3.4. Determination of H_2_O_2_ Concentration Capable of Reducing Cell Viability

The L-929 fibroblasts’ viability was determined after four hours of exposure to various concentrations of H_2_O_2_. The cytotoxicity induced by H_2_O_2_ was dose-dependent. IC_50_ concentration of H_2_O_2_, the concentration capable of reducing the cell viability to 50%, was 50 mM and was used for the following studies. The treatment with H_2_O_2_ on L-929 fibroblasts produced an increase in reactive oxygen species (ROS) levels. Thus, the proliferation of mouse fibroblasts was possibly inhibited owing to their oxidative stress induction, because H_2_O_2_ generally leads to an increase of ROS levels [44]. To set test compounds at concentrations that are harmless to fibroblasts, but could limit the cytotoxicity induced by H_2_O_2_, the effects of various concentrations of all samples on the L-929 fibroblasts’ viability evaluated, and three concentrations of test compounds were selected: 5, 10, and 25 μg/mL.

### 3.5. Determination of the Effect of Pretreatment with Test Compounds on H_2_O_2_-Induced Cell Death

**Figure 3:**
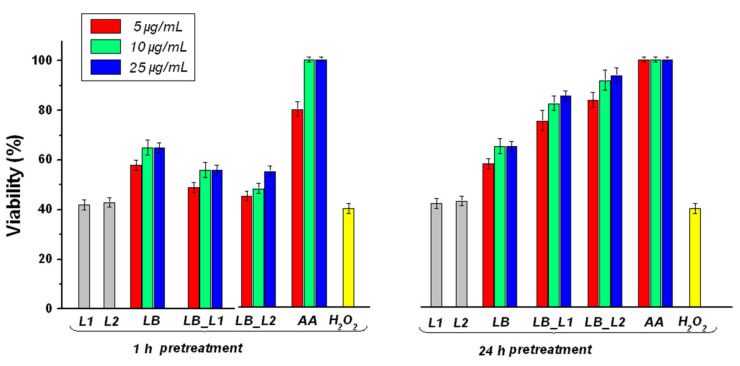
The effect of *L. barbarum* on L-929 cells; pretreatment 1 h or 24 h before applying H_2_O_2_; LB—L. barbarum; LB_L1—liposomes loaded with *L. barbarum*; LB_L2—liposomes loaded with *L. barbarum* (formulation with sodium cholate); AA—acid ascorbic; controls: L1—empty liposomes; L2 —empty liposomes with sodium cholate.

Figure 3 shows the cytoprotective effect of all test compounds on L-929 mouse fibroblast cells. One can observe that, in the case of prolonged pretreatment (24 h) with the test compounds, the cells displayed cytoprotective reactions to oxidative stress. However, the effect was not present in the case of short-term exposed cells (one-hour pretreatment) to loaded liposomes, suggesting that they require a longer time for delivery of polyphenols. A possible explanation for the effect of test compounds on the H_2_O_2_-induced oxidative stress is owing to their phenolic composition. Although all test compounds exhibited a protective effect on L-929 fibroblasts against cytotoxicity induced by H_2_O_2_, the formulation with sodium cholate (LB_L2) provided a better cytoprotective effect than the one without sodium cholate (LB_L1), probably because LB_L2 had a more deformable nature, and thus offers better cell internalization.

Many medicinal plants have antioxidant compounds, mainly polyphenols, which are acknowledged to prevent the degenerative effects produced by oxidative stress, also known as free radical scavengers [45,46,47]. Commonly, in living organisms, ROS is formed in several reactions, produced as by-products during mitochondrial electron transport of aerobic respiration or by oxido-reductase biocatalysts and metal-catalyzed oxidation [48,49,50,51]. Polyphenols, even at low concentrations, provide cell protection, meaning they can inhibit oxidative stress, and thus stop the molecular damage oxidative stress-dependence via several mechanisms [52,53]. The main mechanisms of phenolic compounds as antioxidant agents were described in the literature owing to the existence of some specific groups, namely phenylhydroxyl groups. These groups have the capacity to react with reactive nitrogen species and ROS to form a phenoxy radical thanks to their behavior as hydrogen donors [44]. Moreover, Rise-Evans et al. [54] showed another mechanism for the antioxidant activity of phenolic acids, proving that these plant molecules can relocate or stabilize electrons that are unpaired, catalyze free radicals, and thus possibly behave as oxidative inhibitors. This paper represents the first step for elucidation regarding the antioxidative activity of *L. barbarum* leaves extract. As shown from the literature data, the use of lipid nanoformulation as transporters can aid the process of cell internalization (e.g., endocytosis) of polyphenols, which better interfere with cells at lower concentrations, in comparison with free polyphenols [55,56,57]. The cell membrane is phospholipid-based as well as a liposomal membrane. Thanks to their similar structure, liposomes can pass more easily through biological membranes, thus they can be considered good carriers for molecules (e.g., polyphenols). A free hydro-alcoholic extract, like *L barbarum* hydro-alcoholic extract, finds it harder to enter through biological membranes than the one loaded into a system that consists of the same components as cell membranes. Additionally, some studies have reported the high potential antioxidant effect of polyphenols from the same group of *L. barbarum* leaves against oxidative stress induced by H_2_O_2_ in cerebral glioma cells [55].

### 3.6. Uptake of Liposomes Loaded with L. barbarum by Fibroblasts

**Figure 4:**
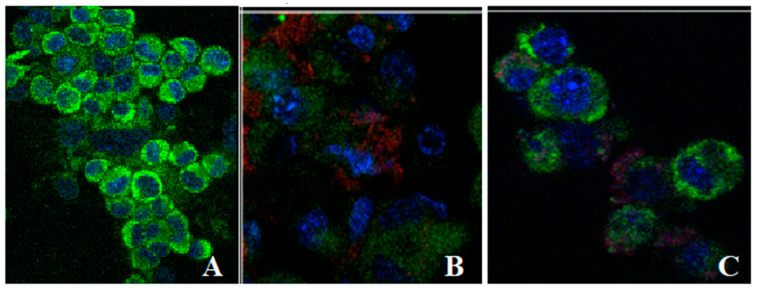
Confocal microscopy images of the uptake of liposomes loaded with *L. barbarum* by L-929 cells. (**A**) Control without liposomes; (**B**) Cells treated with L1_LB; (**C**) Cells treated with L2_LB; the liposomes are labeled with Rhodamine B(red); cell membrane is stained with WGA-AlexaFluor 488 conjugated (green); cell nuclei are stained with Hoechst (blue).

The ability of liposomes loaded with *L. barbarum* to interact with cells was evaluated. Fibroblasts with a cell membrane stained with WGA-AlexaFluor 488 conjugated (green colour) and cell nuclei stained with Hoechst (blue colour) were treated with fluorescent liposomes (red colour). Treated cells were observed using confocal microscopy to assess the trend of internalization and are presented in Figure 4. Confocal microscopy images suggest that liposomes loaded with *L. barbarum* bind to the cell surface and then enter the cell within 24 h. Evidence comes from the localization of rhodamine moiety signals within the cell volume, as revealed by Figure 4, where the red signal of the liposomes was embedded in the green signal of the cell membranes. A tendency of lipsomes to accumulate in the vicinity of the nuclear envelope was noted. L1_LB and L2_LB enter in the cytoplasm, but it is difficult to attribute them to any specific cell organelle.

## 4. Conclusions

*L. barbarum* leaves extract was characterized concerning its total polyphenols content, chemical composition, and antioxidant activity by the Folin–Ciocalteu method, HPLC, and DPPH assay. Data showed the presence of chlorogenic, caffeic, rosmarinic, and gallic acids; total polyphenols content of 18.30 ± 0.011 mg GAE/g plant material; and significant antioxidant activity (EC_50_ = 11.33 ± 0.056 µg/mL). Liposomes containing *L. barbarum* were obtained by the film hydration method combined with sonication and extrusion, and characterized for size, polydispersity, entrapment efficiency, and stability. Loaded liposomes presented 75–85% entrapment efficiency, sizes below 200 nm, narrow polydispersity, and good stability over three months at 4 °C. The lipid nanoformulations lead to an attenuated burst release and produce a slow release of phytocompounds in comparison with the dissolution of *L. barbarum* extract. Moreover, it was investigated whether a pretreatment (one hour or 24 h) with loaded vesicles affected L-929 mouse fibroblasts cytotoxicity induced by H_2_O_2_. A pretreatment (24 h) with liposomal formulations was capable of having a cytoprotective effect on L-929 murine fibroblast cells against cytotoxicity induced by H_2_O_2_. Our findings suggest that liposome encapsulation could be considered a great strategy for the delivery of some antioxidant substances, such as polyphenols, but further investigations are needed.

## Figures and Tables

**Table 1:** Characteristics of liposomes loaded with *L. barbarum*. PDI, polydispersity index; EE, entrapment efficiency.

Sample Code	Particle Size (nm)	PDI	EE (%)	EE—1 Month (%)	EE—2 Months (%)	EE—3 Months (%)
LB_L1	141.6 ± 2.360	0.187 ± 0.001	84.60 ± 2.230	83.75 ± 1.030	81.45 ± 2.150	79.85 ± 1.030
LB_L2	195.9 ± 1.220	0.114 ± 0.001	75.25 ± 1.761	74.53 ± 1.241	72.93 ± 1.171	71.01 ± 1.465
L1	85.4 ± 0.341	0.300 ± 0.003	-	-	-	-
L2	105.2 ± 2.042	0.279 ± 0.005	-	-	-	-

**Table 2:** Parameters of Weibull and Korsmeyer–Peppas models.

Sample Code	Weibull			Korsmeyer–Peppas		
	*a*	*b*	*R* ^2^	*n*	*k*	*R* ^2^
*L. barbarum*	0.937	0.402	0.967	-	-	-
L1_LB	0.541	0.541	0.975	0.215	1.609	0.974
L2_LB	0.158	0.152	0.951	0.180	1.186	0.985

## Data Availability

Data is contained within the article or Supplementary Material.

## Supplementary Materials

The following are available online at https://www.mdpi.com/article/10.3390/nano11081938/s1, Figure S1: RP-HPLC chromatograms of LB_L1 (a) and LB_L2 (b) supernatants after encapsulation of L. barbarum extract; Table S1: Parameters of standard phenolic compounds; Table S2: Polyphenolic compounds concentration entrapped in liposomal formulations.
