# Supplementary material for: Encapsulation of Polyphenols from Lycium barbarum Leaves into Liposomes as a Strategy to Improve Their Delivery
Source: Nanomaterials (Basel). 2021 Jul 28;11(8):1938. doi: 10.3390/nano11081938 (PMC8398605; doi:10.3390/nano11081938)
Supplement: Supplementary file 1 [file nanomaterials-11-01938-s001.zip › nanomaterials-1285562 supplementary.pdf]

Supplementary Materials: Encapsulation of Polyphenols from *Lycium Barbarum* Leaves into Liposomes as a Strategy to Improve Their Delivery

Ramona-Daniela Păvăloiu [1,2,*], Fawzia Sha'at [1,2], Georgeta Neagu [1], Mihaela Deaconu [2], Corina Bubueanu [1], Adrian Albulescu [1,3], Mousa Sha'at [4], and Cristina Hlevca [1]

[1] National Institute for Chemical-Pharmaceutical Research & Development—ICCF Bucharest, Vitan Av. nr. 112, 3rd district, 031299, Bucharest, Romania; fawzya.shaat@gmail.com (F.S.); georgetaneagu2008@gmail.com (G.N.); cbubueanu@gmail.com (C.B.); rockady2@gmail.com (A.A.); crhlevca@gmail.com (C.H.)

[2] Department of Inorganic Chemistry, Physical-Chemistry & Electrochemistry, Faculty of Applied Chemistry and Materials Science, University "Politehnica" of Bucharest, Gheorghe Polizu Street nr. 1–7, 1st district, 011061 Bucharest, Romania; mihaelladeaconu@gmail.com

[3] Department of Molecular Virology, Stefan S. Nicolau Institute of Virology, Mihai Bravu Av. nr. 285, 3rd district, 030304 Bucharest, Romania

[4] Department of Pharmaceutical Technology, Faculty of Pharmacy, University of Medicine and Pharmacy Grigore T. Popa, Universitatii Street. nr. 16, 700115 Iasi, Romania; mousa.shaat1@gmail.com

* Correspondence: pavaloiu.daniella@gmail.com; Tel.: +40-0728-397-515

1. Characterization of L. barbarum extract

Table S1. Parameters of standard phenolic compounds

Compound	*RT* ± SD, min	λ_{max}, nm	Calibration curve	R^2
Gallic acid	3.82 ± 0.10	271	y=8371.14*x-1207.10	0.9993
Protocatechuic acid	7.32 ± 0.09	360	y=9007.30*x-1186.03	0.9997
Caftaric acid	11.09 ± 0.04	329	y=3741.63*x-1004.52	0.9992
Catechin hydrate	12.79 ± 0.27	279	y=1704.55*x-1484.97	0.9974
Chlorogenic acid	13.54 ± 0.26	326	y=7634.01*x-1686.39	0.9984
Vanillic acid	15.27 ± 0.03	261	y=8457.44*x-1216.69	0.9999
Caffeic acid	15.53 ± 0.22	323	y=13188.1*x-1501.42	0.9989
Syringic acid	16.89 ± 0.03	275	y=6929.03*x-667.27	0.9997
(-)-Epicatechin	17.82 ± 0.04	278	y=1585.49*x-273.15	0.9998
p-Coumaric acid	22.29 ± 0.24	309	y=18220.6*x-1642.10	0.9946
trans-Ferulic acid	25.51 ± 0.05	323	y=3135.95*x-769.69	0.9999
Ellagic acid dihydrate	25.62 ± 0.06	252	y=5128.27*x-577.18	0.9996
Rutin hydrate	26.50 ± 0.02	355	y=3813.02*x-838.90	0.9985
Chicoric acid	29.37 ± 0.05	330	y=10560.6*x-1939.03	0.9975
Myricetin	31.95 ± 0.13	373	y=9150.32*x-1464.36	0.9991
Rosmarinic acid	31.73 ± 0.10	330	y=7282.31*x-633.29	0.9988
trans-Resveratrol	33.43 ± 0.11	307	y=17601.1*x-2585.25	0.9989
Quercetin	34.91 ± 0.05	371	y=9898.83*x-723.17	0.9994
Kaempferol	35.94 ± 0.03	367	y=10549.7*x-1296.00	0.9981

2. Characterization of liposomes loaded with L. barbarum

2.1. HPLC analysis of supernatants

After the extract was encapsulated in liposomes, each supernatant was separated and analyzed through RP-HPLC to quantify the polyphenolic compounds. The results are presented in Figure S1 and Table S2.

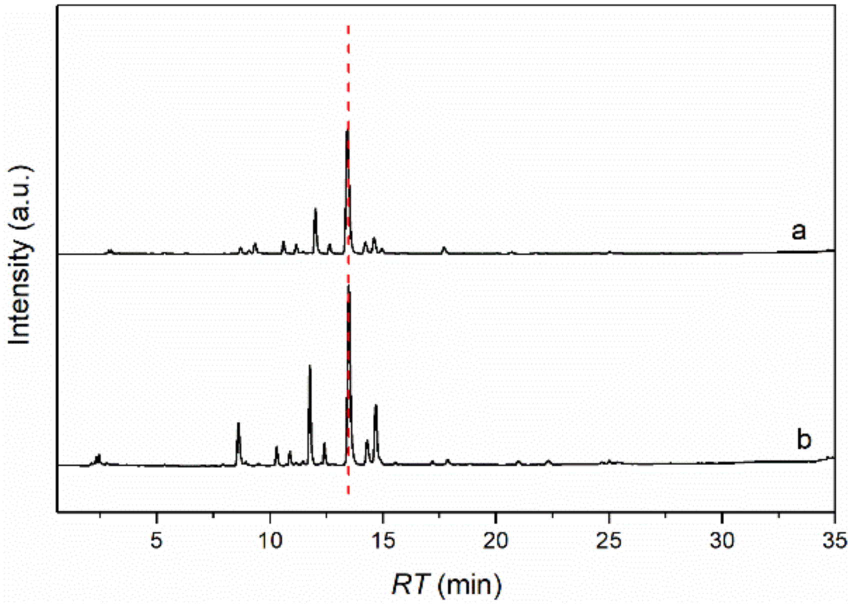

Figure S1. RP-HPLC chromatograms of LB_L1 (a) and LB_L2 (b) supernatants after encapsulation of *L. barbarum* extract

Table S2. Polyphenolic compounds concentration entrapped in liposomal formulations

**Polyphenolic compound**	**RT, min**	**LB_L1 supernatant concentration, mg/g extract**	**LB_L2 supernatant concentration, mg/g extract**	**LB_L1 concentration, mg/g extract**	**LB_L2 concentration, mg/g extract**
Gallic acid	3.85	0.060 ± 0.001	0.059 ± 0.001	0.171 ± 0.001	0.172 ± 0.001
Chlorogenic acid	13.55	6.682 ± 0.001	3.252 ± 0.001	12.058 ± 0.001	15.488 ± 0.001
Caffeic acid	15.50	ND	0.032	1.773 ± 0.002	1.741 ± 0.001
Rosmarinic acid	31.71	ND	ND	0.108 ± 0.001	0.108 ± 0.001

* The quantity of encapsulated polyphenols was calculated by subtracting the amount of polyphenols used in initial formulation from amount of unbound polyphenols present in supernatant obtained after centrifugation of liposomes

** ND - not detected
